# Shedding light on the expansion and diversification of the Cdc48 protein family during the rise of the eukaryotic cell

**DOI:** 10.1186/s12862-016-0790-1

**Published:** 2016-10-18

**Authors:** Nickias Kienle, Tobias H. Kloepper, Dirk Fasshauer

**Affiliations:** 1 Département des neurosciences fondamentales, Université de Lausanne, Rue du Bugnon 9, CH-1005 Lausanne, Switzerland; 2 Sir William Dunn School of Pathology, Research Group Cell Biology of Intercellular Signaling, University of Oxford, South Parks Road, Oxford, OX1 3RE UK

**Keywords:** Organelles, Compartmentalization, Eukaryogenesis, LECA, SELMA, Vesicle trafficking, AAA+ proteins, NSF

## Abstract

**Background:**

A defining feature of eukaryotic cells is the presence of various distinct membrane-bound compartments with different metabolic roles. Material exchange between most compartments occurs via a sophisticated vesicle trafficking system. This intricate cellular architecture of eukaryotes appears to have emerged suddenly, about 2 billion years ago, from much less complex ancestors. How the eukaryotic cell acquired its internal complexity is poorly understood, partly because no prokaryotic precursors have been found for many key factors involved in compartmentalization. One exception is the Cdc48 protein family, which consists of several distinct classical ATPases associated with various cellular activities (AAA+) proteins with two consecutive AAA domains.

**Results:**

Here, we have classified the Cdc48 family through iterative use of hidden Markov models and tree building. We found only one type, Cdc48, in prokaryotes, although a set of eight diverged members that function at distinct subcellular compartments were retrieved from eukaryotes and were probably present in the last eukaryotic common ancestor (LECA). Pronounced changes in sequence and domain structure during the radiation into the LECA set are delineated. Moreover, our analysis brings to light lineage-specific losses and duplications that often reflect important biological changes. Remarkably, we also found evidence for internal duplications within the LECA set that probably occurred during the rise of the eukaryotic cell.

**Conclusions:**

Our analysis corroborates the idea that the diversification of the Cdc48 family is closely intertwined with the development of the compartments of the eukaryotic cell.

**Electronic supplementary material:**

The online version of this article (doi:10.1186/s12862-016-0790-1) contains supplementary material, which is available to authorized users.

## Background

In contrast to prokaryotes, which generally consist of a single intracellular chamber surrounded by a plasma membrane, eukaryotic cells are subdivided into various functionally distinct internal membrane-bounded compartments, including the nuclear membrane, the endoplasmic reticulum (ER), the Golgi apparatus, lysosomes, endosomes, and the cell membrane. Material exchange between compartments of this vast endomembrane system occurs by membrane-enclosed vesicles that bud off from one membrane and specifically fuse with an acceptor compartment after moving along cytoskeletal tracks.

The central machine involved in the vesicle fusion process in each trafficking step is composed of soluble *N*-ethylmaleimide-sensitive factor attachment protein receptor (SNARE) proteins. These tail-anchored membrane proteins operate via a fundamental mechanism: their sequential assembly into tight membrane-bridging complexes pulls the two membranes together. Their activity is orchestrated by various other conserved factors including Sec1/Munc18 (SM), Rab, and tethering proteins [1–5]. During different vesicle trafficking steps, the vesicle docking and fusion process is carried out by distinct sets of these factors. This suggests that they arose by duplication and diversification of a prototypic vesicle fusion machinery. This, in turn, implies that the proto-eukaryotic cell was already equipped with the various compartments and the vesicle transport machinery found in contemporary cells [6–9]. Intriguingly, no direct orthologs of SNARE proteins have been identified in prokaryotes yet.

Breaking apart SNARE complexes requires the activity of the ATPase *N*-ethylmaleimide-sensitive factor (NSF), which is mostly present as singleton in all eukaryotes. This essential factor was originally discovered on the basis of its role in ER-Golgi trafficking [10]. Together with its soluble NSF attachment protein (SNAP) cofactor, NSF hydrolysizes ATP to disassemble SNARE complexes and thus releases individual SNARE proteins for another round of fusion [11, 12]. From today’s perspective, it therefore appears that the evolution of the vesicle fusion mechanism required a disassembly ATPase in order to refuel the SNARE engine. But where did the disassembly machinery come from?

When searching for the origins of the disassembly machinery, one does not need to enter unknown territory, as NSF is one of the founding members of the large superfamily of ATPases associated with various cellular activities (AAA+) (reviewed in [13–18]) that can be found in all three domains of life. One characteristic of the AAA+ superfamily is a conserved ATP-binding domain, the so-called AAA domain. AAA domains form hexameric rings that are essential parts of various machines whose fundamental function is to unfold proteins. During this process, AAA domains undergo large movements driven by ATP hydrolysis. Early phylogenetic surveys of the AAA+ superfamily using cluster approaches have shown that NSF and its relatives belong to the clade of classical AAA proteins, which also contain proteasome subunits, metalloproteases, meiotic ATPases, and BCS1 [19–21]. In contrast to other classical AAA proteins, which usually have only one AAA domain, the relatives of NSF possess two AAA domains arranged in a line, termed the D1- and D2-domain. This family is sometimes referred to as the Type-II AAA protein [22, 23]). The D1-domain of NSF is thought to drive SNARE disassembly by ATP hydrolysis, whereas the D2 domain is involved nucleotide-dependent hexamerization. NSF also has an *N*-terminal domain that interacts with the SNARE complex and the SNAP adaptor protein [24–26]. NSF shares this domain architecture with Cdc48 (also known as p97 or valosin-containg protein (VCP)), another founding member of the protein family [27]. Initially, it was thought that Cdc48 and NSF had overlapping functions, as both factors are involved in the re-assembly of the Golgi apparatus, the ER, and the nuclear envelope after mitosis, but subsequently, it was discovered that Cdc48 is involved in a broad spectrum of seemingly unrelated cellular activities (e.g. [28–31]). Its functional diversity is determined by differential binding to a large number of adaptor proteins. Its function is best understood during ER-associated protein degradation (ERAD) by the ubiquitin-proteasome system. Other founding members of the family are the proteins Pex1 and Pex6, which play a key role in peroxisomal matrix protein import [32, 33]. The other known family members are involved in the export of ribosome subunits from the nucleus (nuclear VCP-like (NVL), also known as smallminded in *Drosophila*, as mac-1 in *Caenorhabditis elegans*, or as Rix7 in *Saccharomyces cerevisiae*) and cytosolic ribosome maturation (Spaf, also referred to as SPATA5 and Drg1 in *Saccharomyces cerevisiae*) [34], and regulation of nucleosome density (Yta7 (referred to as ATAD2 in animals)) [35]. Intriguingly, many pathways of these important factors converge on selective proteolysis by the ubiquitin-proteasome system. Here, we will refer to these factors as the Cdc48 family. The different family members power a wide range of important cellular processes that, in many cases, take place in different compartments of the eukaryotic cell. It was therefore suggested early on that NSF had been derived from a versatile protein unfolding factor during the emergence of the eukaryotic endomembrane system [19, 20, 36]. As only one family member, Cdc48 (also referred to as VCP-like ATPase of *Thermoplasma acidophilum* (VAT)) is present in archaea and in some eubacteria [30]), a detailed history of the Cdc48 family might thus provide an opportunity to glance at a period during which the organizational complexity of the eukaryotic cell evolved.

Although the Cdc48 family has successively emerged as a single clade in earlier phylogenetic studies [19, 20, 22, 36–41], the exact relationship and taxonomic distribution of the different members of the family are not entirely clear yet. Here, we take a fresh look at the ramifications of the tree of this protein family, taking advantage of the enormous growth of sequence data over the last years. For our investigation, we have iteratively built and refined hidden Markov models (HMMs) for these subfamilies. This enabled us to gather an exhaustive sequence collection and to construct comprehensive evolutionary trees for this family. We have established an Cdc48 Database web server to make the classifiers and full analysis available. Moreover, we used the large sequence collection to determine sequence-specific properties for each family member, providing an important basis for future structure-function studies on these proteins.

## Results & discussion

### A HMM-based classification of the Cdc48 family AAA domains

We started by collecting approximately 600 AAA domain-containing sequences of established Cdc48 family members [19]. In general, each of these sequences contains two consecutive AAA domains, referred to as the D1- and D2-domains. From an initial alignment, we extracted the conserved D1- and D2-domain regions, joined them into one large D-domain alignment, and performed a phylogenetic analysis as well as a similarity-based analysis using CLuster ANalysis of Sequences (CLANS) [42]. From these analyses, we were able to define the hierarchical relationship of the prominent domain subgroups. For each of these subgroups, we created a HMM and searched various genome databases for AAA domain-containing sequences. With the expanded data set we repeated the analysis and refined the set of specific HMMs. This process was repeated until no further improvement in the classification could be achieved (see Additional file 1: Figure S1 for the HMM statistics). In total, we collected 3911 sequences containing 7639 motifs from 527 eukaryotic and 235 archaeal species.

### The last eukaryotic common ancestor (LECA) contained a diverse set of double-ring AAA ATPases

**Fig. 1:**
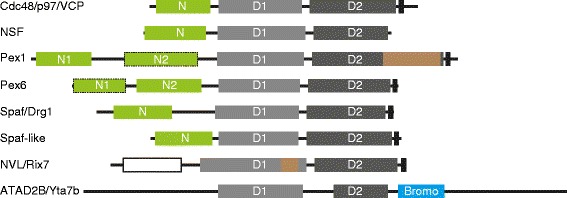
Domain organization of the different human members of the Cdc48 family. The tandem D-domains, D1 and D2, are shown in grey. *N*-domains with φβ double barrel fold are shown in *green*; the deviating *N*-terminal domain of nuclear VCP-like (NVL) is shown as a *white box*. The putative second *N*-domains of Pex1 (N2) and of Pex6 (N1) are highlighted by *dashed boxes*. The larger inserts into the D1-domain of NVL and the D2-domain of Pex1 are shown in *brown*. The tail helices at the *C*-terminal end of D2-domains are indicated as *black boxes*. The bromodomain in the D2-domain of Yta7 is shown in *blue* and is located right after the *N*-terminal subdomain containing the Rossman fold. Note that vertebrates generally possess two Yta7 homologs, referred to as ATAD2 and ATAD2B; only one of the two human Yta7 variants is shown. Note that the detailed arrangement of the secondary structural elements of the two D-domains of Cdc48 is given in Additional file 3: Figure S2. The novel family member Spaf-like has been discovered in screens for chronic kidney disease [113, 114] and has also been found in several interactome studies (e.g. [64, 115–117], suggesting that it plays a role in selective protein degradation. Spaf-like constitutes a distinctive branch that has been not recognized clearly in earlier surveys, probably because this factor is present in only a few eukaryotic lineages. Generally, its domain structure is similar to that of Cdc48. However, as noted earlier [19], Spaf-like from *Arabidopsis thaliana* has no *N*-domain and contains a transmembrane region at its *C*-terminal end. As more sequence information is now available, we found that a *C*-terminal transmembrane region is shared by all Spaf-like from core eudicots, suggesting that the membrane anchor was gained in this lineage. By contrast, the loss of the *N*-domain appears to have occurred much earlier in plants, as we did not find it in most plants, apart from the green algae group Mamiellales (*Ostreococcus*, *Micromonas*). It cannot be excluded, however, that the absence of this domain in some species is caused by incomplete sequence assembly. Recurrently, we came across a few more diverged double-ring AAA ATPase sequences that formed longer branches in our phylogenetic trees and that appear to be more closely related to Cdc48 than to any other member of the family. As we discovered these sequences in several diverse lineages, including heterokonts, amoebozoa, a few green algae, and basal fungi, but not in animals, they might constitute another basal family member. We named this factor Cdc48-like, but cannot currently exclude the possiblity that Cdc48-like is a collection of more diverged Cdc48 variants that group together because of long-branch attraction

Our exhaustive classification revealed that in eukaryotes, the Cdc48 family consists of eight different types, whereas generally only one type is found in prokaryotes. An overview of the domain structure of the different eukaryotic family members is given in Fig. 1. In addition to the seven already established factors (Cdc48, NSF, Pex1, Pex6, SPAF, NVL, and Yta7) [19] we came across an additional family member. This is the product of a gene called spermatogenesis associated 5-like 1 (SPATA5L1). Little is known about its cellular role. As it is closely related to Spaf (SPATA5), we will refer to the factor as Spaf-like. Generally, the eight different Cdc48 family members are present in most eukaryotic lineages (Additional file 2: Table S1), suggesting that these proteins were present in the last eukaryotic common ancestor (LECA), supporting the view that this organism was rather complex [6]. This basic set has not been expanded in all major eukaryotic phyla, although some factors have been duplicated and some have been lost, as will be outlined below.

### Modifications of the D-domains in different family members

By and large, the domain arrangement of several family members have remained similar to that of Cdc48, comprising an *N*-domain and two distinct AAA domains in tandem. A detailed arrangement of the secondary structural elements of Cdc48 is given in Additional file 3: Figure S2. Some family members have a modified domain arrangement, however. For example, NVL and Yta7 carry novel *N*-domains. In addition, Yta7 also possesses a bromodomain within its second D-domain. Bromodomains are interaction modules that specifically recognize ε-*N*-lysine acetylation motifs, a modification found mostly in histones. More subtle changes occurred in the D-domains of other family members. For example, the D1-domain of NVL has an insert approximately 50 amino acids long before the terminal helix of the domain [34]. Furthermore, we detected a large insert after Helix α7 of the D2-domain of Pex1. This insert might be flexible, as it was not resolved in the crystal structures of Cdc48 [43] and NVL (PDB ID: 2X8A).

### Phylogenetic relationships within the Cdc48 family

In order to resolve the phylogenetic relationships of the Cdc48 protein family, we first calculated the phylogenetic trees of the individual D-domains. To reduce bias and to minimize computational effort, we generated a reduced list of species that represent all known major lineages of the eukaryotic phylum. To root the tree, we included several Archaea sequences (Additional file 4: Table S2). We used all the individual D-domains of different Cdc48 family members from the selected species. Eventually, we removed the D2-domain of Yta7, which contains a bromodomain, from our calculation, as it formed a long branch. In fact, we did not consider the rudimentary D2-domain of Yta7 for our phylogenetic analysis in the following.

**Fig. 2:**
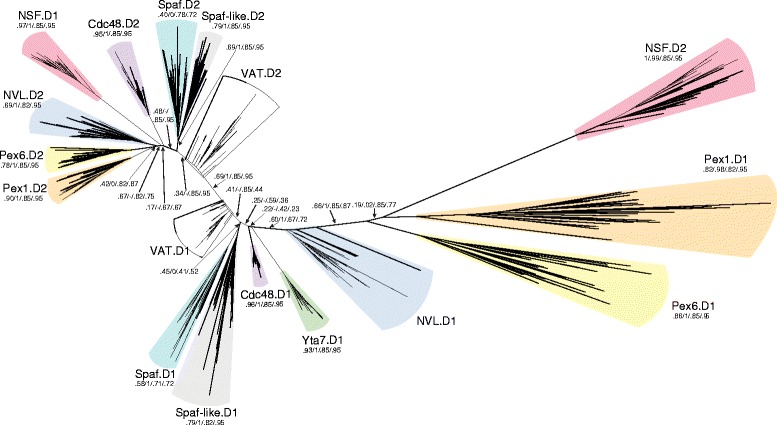
Evolutionary tree of the individual AAA domains of the different Cdc48 family members. The tree was constructed from the individual AAA domains (D1- and D2-domains) of the Cdc48 family using a selection of 48 eukaryotic and 26 archaeal species, accounting for a total of 687 AAA domains. The representative species are listed in Additional file 2: Table S2. The different family members are highlighted in different colors, while D1- and D2-domains of the same protein have the same color. Statistical support values (likelihood-mapping/IQ-TREE support/RAxML support/PhyML support) are given at selected inner edges. Most AAA domains form short branches and split into a D1 and a D2 subtree, in which the two domains of all archaeal Cdc48 are located close to the center of the tree, probably reflecting the fact that the eukaryotic family members are derived from a primordial VAT [19]. However, in the tree all archaea sequences, even from the recently found Lokiarchaeota, are well-separated from eukaryotic family members. The two more divergent D1-domains of the peroxins and the D2 domain of the N-ethylmaleimide sensitive factor (NSF) form long branches. Notably, the D2-domain of NSF is located in the D1 subtree, whereas the D1-domain of NSF is found in the D2 subtree. A similar pattern had been observed in earlier studies and it has been suggested that the two domains of NSF have been swapped during evolution [118]. Given the generally conserved architecture of the protein family, this scenario is not very likely [39]. It is much more probable that this branching pattern is caused by long-branch attraction. In fact, when we included the incomplete, long-branching D2-domain of Yta7, the branching pattern of the two NSF domains changed (Additional file 11: Figure S7)

**Fig. 3:**
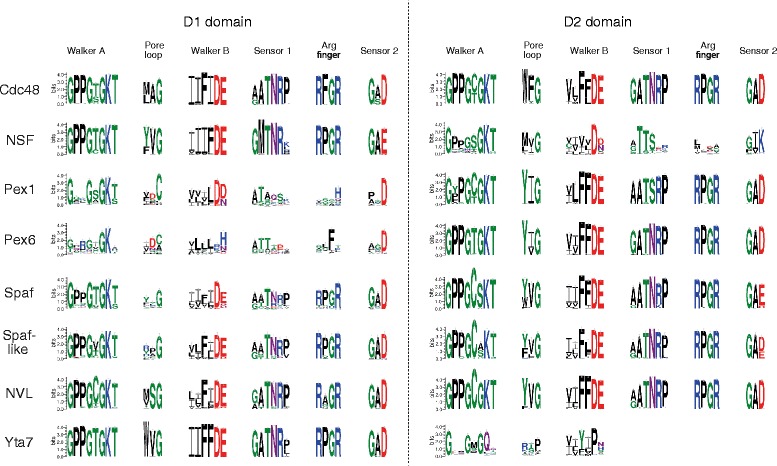
WebLogo representation of the key sequence elements of the Cdc48 family. Sequence logos were generated from alignments of the D-domains of different Cdc48 family members using the WebLogo software [119]. Alignment contained more than 500 eukaryotes. The key regions involved in nucleotide binding and hydrolysis and the pore loop as defined by [13, 14, 18] are shown. The overall height of a stack indicates the sequence conservation at a certain position; the height of the symbols within the stack indicates the relative frequency of each amino acid at that position. The sequence logo of the entire alignment is provided in Additional file 12: Figue S8

Consistent with earlier surveys [19, 20, 22, 36–39], the tree revealed that most of the D-domains are well conserved; in particular, the two D-domains of Cdc48 are highly conserved and show surprisingly little sequence variation (Fig. 2). Most of the different D-domains from eukaryotic proteins form distinct, well-supported branches in the tree, indicating clear speciation. However, some AAA domains show especially strong speciation. These are the D1-domains of the two peroxins Pex1 and Pex6 and the D2-domain of NSF in particular. Notably, these are the family members that seem to have departed considerably from the original role of the family. The degeneration of these D-domains has been noted in the earlier surveys [19, 20, 22, 36–41], but our broad phylogenetic sampling allowed us to inspect this aspect in greater detail. We used sequence logos to depict the conservation pattern of the key regions of the two D-domains of the different Cdc48 family members (Fig. 3). This revealed that in the D1-domains of Pex1 and Pex6, several elementary motifs of the AAA domain (e.g. the Walker A and B motifs, which are key elements for nucleotide binding) have severely degenerated (Fig. 3). In addition, the arginine finger regions of the D1-domains of Pex1 and Pex6 are not conserved. This region usually comprises two spaced arginines that interact with the nucleotide-binding pocket of the neighboring subunit. It has been demonstrated that the D1-domains do not contribute to the ATPase activity of the Pex1-Pex6 complex, but might function in hexamerization of the complex [44].

### The D2-domain of NSF lacks a tail helix

Several key motifs of the D2-domain of NSF have degenerated in a similar manner. In particular, a marked difference between NSF and other Cdc48 family members can be seen in the Sensor 2 region of the D2-domain of NSF (Fig. 3). The Sensor 2 region is located at the base of Helix α7 in the *C*-terminal α-subdomain of AAA+ proteins (Additional file 3: Figure S2). Most AAA+ proteins possess a conserved GAR motif in this region. The arginine of this motif contacts the bound nucleotide and contributes to ATP hydrolysis. In classical AAA proteins, the positively charged arginine in the GAR motif is changed to an aspartate (GAD) [14] or, more rarely, to a glutamate carrying a negative charge. Indeed, we generally found the GAD motif to be highly conserved in the D1- and D2-domains of the Cdc48 family. Intriguingly, in the structure of Cdc48, the aspartate does not face the nucleotide-binding site [43]. In the D1-domain, the Sensor 2 aspartate contacts a conserved stretch at the base of the D1-D2 linker and might be important for communication between the two D-domains (Additional file 5: Figure S3). In the D2 -domain, the Sensor 2 aspartate might help to position the tail helix of Cdc48 [43, 45].

Interestingly, the third position in the Sensor 2 motif of the D2-domain of NSF is held by a lysine or, more rarely, by an arginine (Fig. 3). This lysine contacts the γ-phosphate of the ATP in the structure of the D2-domain of NSF [46, 47], somewhat comparable to the orientation of the arginine in the GAR motif of other AAA+ proteins. But why was the conserved Sensor 2 aspartate of the Cdc48 family maintained in the D1-domain of NSF but not in its D2-domain? The reason might be that NSF has lost the C-terminal tail helix and thus did not require a Sensor 2 aspartate in the D2-domain to position this helix. But what is the role of the tail helix? The tail helix of Cdc48 contains several bulky side chains that face the Sensor 1 loop of the D2-domain. It has been suggested that this interaction “pushes” the Sensor 1 loop towards the nucleotide binding site of the D2-domain [43]. In fact, in the Cdc48 structure, a tyrosine, Y755, in the tail helix directly contacts N624 of the Sensor 1 loop. The tail helix thus might be important for coordinating the conformational changes in the D2-domain during ATP hydrolysis. We found the tail helix with a central tyrosine residue to be conserved in all Cdc48 family members (Additional file 6: Figure S4) except NSF and Yta7. The preservation of the tail helix in most Cdc48 family members suggests that it plays an important functional role in these double-ring AAA ATPases. The tail helix seems to be already present in the archaeal VAT and might thus represent a molecular characteristic of this family that has not been maintained in NSF and Yta7.

### Conservation of the linker regions

It is thought that in NSF, the D2 is crucial for nucleotide-dependent hexamerization, whereas its D1-domain is catalytically active. By contrast, the D2-domain of Cdc48 has greater ATPase activity than its D1 domain. ATP hydrolysis by the D2-domain is thought to drive the large conformational change that pulls the unfolded substrate, although the precise molecular mechanism of this machinery is still debated. The D1-domain of Cdc48 is thought to contribute additional activity at higher temperatures. The large conformational changes need to be well orchestrated in double-ring AAA ATPases to ensure that the substrate is passed on from the *N*-terminal domain to the tandem D-domains. In Cdc48, the ubiquitinated substrate is often transferred to the proteasome for degradation. This is likely to be different for NSF, which disassembles SNARE complexes in order to reuse their constituents. For this process, NSF might not need to pull the substrate along its D2-domain and hence the D2-domain may have acquired a more static role. This functional swap between the two domains of NSF probably led to adjustments in the other domains as well. Some changes can be seen in the linker regions, for example. The linker regions have been studied intensively, as they play an important role in the interdomain communication of Type II AAA+ ATPases (e.g. [45, 48–53]). The linker between the D1- and D2-domain contains a conserved motif containing two glycines right before Helix α0. This motif participates in nucleotide binding but it is not well conserved in NSF (Additional file 5: Figure S3). A homologous stretch is present in the linker between the *N*-domain and the D1-domain, supporting the idea that a D-domain including the linker region was duplicated at the emergence of the Cdc48 family. This linker region is conserved in some subfamilies, particularly in Cdc48; however, only the two glycines are highly conserved throughout the entire family (Additional file 7: Figure S5). Intriguingly, this region has undergone a large conformational change in Cdc48 during ATP hydrolysis [54–56].

### A duplication of the *N*-domain has occurred in peroxins

Several Cdc48 family members share a homologous kidney-shaped *N*-domain consisting of two smaller subdomains [57–61]. This corroborates the idea that all Cdc48 family members originated from a common ancestor, although NVL and Yta7 have adopted novel *N*-domains. To complement our survey, we examined the evolutionary history of this domain as well. Taking advantage of our sequence collection, we made specific HHMs for each family member with a canonical *N*-domain. As well as the proteins listed above, our list of family members that have an obvious canonical *N*-domain included Spaf, Spaf-like, and Pex6. We then calculated a phylogenetic tree for all well-conserved *N*-domains (Additional file 8: Figure S6). Interestingly, although the *N*-domain of Cdc48 is highly conserved, the *N*-domains of the other family members diverged more. In general we noted that, compared to the D-domains, the *N-* domains are generally less conserved. This agrees with the fact that we were unable to build a specific HMM that incorporates all *N*-domains. Possibly, the *N*-domains of the different family members adapted to different substrates when they adapted new functions.

We noted that the canonical *N*-domains of Pex1 [60] and Pex6 are located in different *N*-terminal sections (Fig. 1). In fact, the two peroxins have a rather extended *N*-terminal region compared to other family members. Whereas the *N*-domain of Pex1 is situated in the most *N*-terminal portion, the *N*-domain of Pex6 is found more C-terminally, close to the D1-domain. Interestingly, for some peroxin sequences, our HMMs detected a second *N*-domain region, although this had very low E-values. When we evaluated different secondary structure predictions for both peroxins, we found that both peroxins contained a second stretch with several secondary structure elements in their *N*-terminal regions. Very probably, these additional stretches are highly diverged *N*-domains or remnants thereof. It was recently confirmed that the two peroxins possess two consecutive *N*-domains [44, 62]. The presence of tandem *N*-domains corroborates the notion that the two peroxins arose from a common ancestor.

### Early duplications within the Cdc48 family before the LECA

**Fig. 4:**
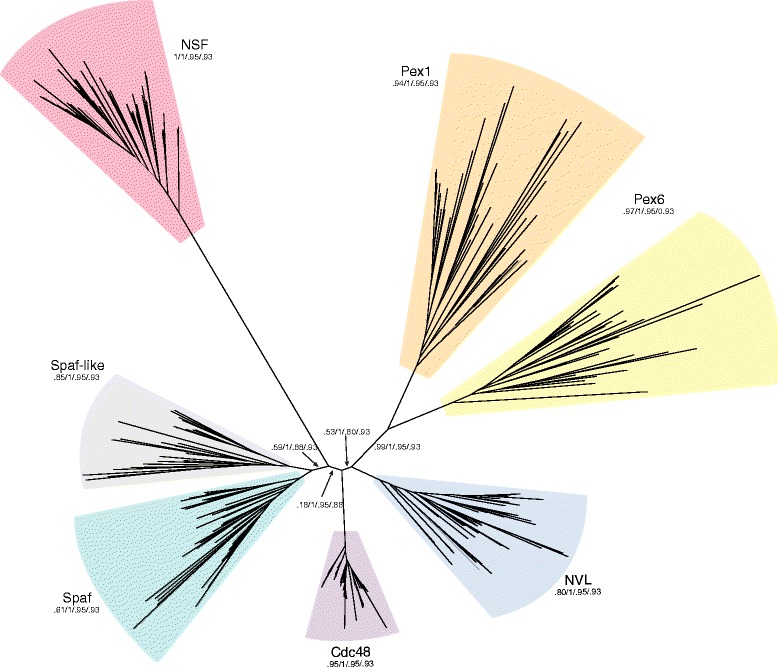
Evolutionary tree of the combined D1- and D2-domains for different Cdc48 family members. The tree was calculated using an alignment of concatenated D1- and D2-domains of 48 eukaryotic species comprising 270 sequences. The tree shows a distinct separation of the individual Cdc48 family members (highlighted in different colors as in Fig. 2). Statistical support values are annotated at selected inner edges. Note that the most divergent member, Yta7, was omitted

The scenario in which Pex1 and Pex6 arose from a common ancestor is corroborated by the fact that both D-domains of peroxins usually branch together (Fig. 2). This pattern became even more apparent when both D-domains were combined for tree calculation (Fig. 4). This pattern suggests that they arose by gene duplication before the rise of the LECA. This notion is consistent with the observation that the two factors work together in a hetero-hexameric ring with alternating subunits [51, 63]. Thus, the ancestral Pex machinery was originally a homo-hexameric ring. Later the two subunits might have shared labor within the same complex.

It also can be seen that the two factors Spaf/Drg1 and Spaf-like are on one branch of the phylogenetic tree, suggesting that they had a common ancestor. However, it is not known whether the two factors, like Pex1 and Pex6, can work together. On the one hand, this scenario is unlikely, as Spaf-like has been lost in several lineages (e.g. in fungi) while Spaf/Drg1 has been maintained more widely. On the other hand, we noted that both factors were found to interact in a large-scale screen [64] and it should be tested whether both factors work in cytoplasmic ribosome maturation.

Another putative pre-LECA duplication might have led to the rise of the two factors Cdc48 and NVL. The D-domains of these two factors are conserved, but they comprise two structurally different types of *N*-domains. Although several family members have an αβ *N*-domain, the *N*-terminal region of NVL is mostly α-helical. Interestingly, the *N*-terminal domain of NVL contains a nuclear localization signal that steers the factor into the nucleus to work in early steps of ribosome biogenesis, whereas Cdc48 is found in the cytosol and the nucleus.

As mentioned above, the LECA possessed at least eight distinct members of the Cdc48 family. If we take the internal duplications into account, this set can be arranged into five distinct basic types (NSF, Yta7, Pex1/6, Cdc48/NVL, and Spaf/Spaf-like) that are likely to have worked in an earlier phase of the evolution of the eukaryotic cell. According to our analysis, NSF, Pex1/6, and Yta7 have diverged more, probably to adapt to novel functions, whereas Cdc48/NVL and Spaf/Spaf-like remained more archetypical.

The internal duplications within the Cdc48 family must have occurred in earlier evolutionary stages of the eukaryotic cell. For example, it has been recognized that the machinery for peroxisomal protein import is homologous to the machinery that degrades ER-associated proteins (ERAD) [33, 40, 65, 66]. In both processes, members of the Cdc48 family play a key role but other factors also seem to have been duplicated. Both machineries move ubiquitinylated proteins across a membrane. Whereas Cdc48 fuels the re-translocation of misfolded proteins of the ER into the cytosol, where they are eventually degraded by the proteasome, the peroxin ring drives the import of peroxisomal matrix proteins. Our phylogenetic analysis corroborates the idea that the machineries for ERAD and peroxisomal protein import had a common ancestor, although the peroxins were co-opted for a new function; they split into two different subunits, Pex1 and Pex6, which work together in one machine. It is likely that peroxisomes are off-shoots of a primordial ER-like compartment that might have existed in a pre-LECA eukaryote. This is supported by the fact that peroxisomes can form *de novo* from the ER [67–69].

### Variations of the LECA set in different eukaryotic lineages: defining a minimal repertoire

To detect losses and gains in extant eukaryotic lineages, we calculated individual phylogenetic trees for each of the family members. We found that the Cdc48 family member Spaf-like has been lost independently in several lineages. For example, although we found Spaf-like in the genome of a few basal fungi, it has been lost in all other fungi including *S. cerevisiae*. We were unable to detect Spaf-like in all the alveolates that we inspected. Because of the lack of functional data, it is currently impossible to correlate these losses to a change in a particular cellular process. The same can be said for the conspicuous absence of the chromatin-interacting factor Yta7 in alveolates, for example.

As expected, drastic losses occurred in parasitic lineages. An account is given in Table 1. However, a parasitic lifestyle did not necessarily lead to a loss of these factors, since some parasitic species have a complete (e.g. oomycetes) or an almost complete repertoire of double-ring AAA factors (e.g. euglenoids). Intriguingly, we found the most reduced set of Cdc48 family members we found in *Giardia* and Microsporidia. Their genomes seem to encode only for Cdc48, NSF, and NVL. Notably, we found these three factors to be present in every eukaryotic genome, suggesting that they represent the minimal set for eukaryotic cell function, while the other factors appear to be expendable under certain conditions.

**Table 1:** Repertoire of the Cdc48 family members in selected parasitic lineages and species. A filled black circle indicates the presence of the particular family member in the genome of the organism. For comparison, the repertoire of Homo sapiens is given as well

Species/Lineages	Cdc48	NSF	Pex1	Pex6	Spaf	Spaf-like	NVL	Yta7
*Blastocystis hominis*	•	•					•	•
*Plasmodium*, *Babesia*, *Theileria* ^a^	••	•			•		•	
*Oomycetes* ^a^	••	•	•	•	•	•	•	•
*Trichomonas vaginalis*	•	•			•	•	•	
*Leishmania*, *Trypanosoma*	•	••	•	•	•		•	•
*Giardia*	•	•					•	
*Microsporidae* ^b^	•	•					•	
*Entamoeba*	•	•			•	•	•	
*Oikopleura dioica*	•	•			•		•	•
*Trichuris*, *Trichinella* ^c^	•	•			•		•	
Parasitic *Platyhelminthes* ^d^	•	•					•	•
*Homo sapiens*	•	•	•	•	•	•	•	••

^a^Based on repertoire of the species listed in Additional file 9: Table S3

^b^Based on the genomes of *Edhazardia aedis*, *Enterocytozoon bieneusi*, *Encephalitozoon cuniculi*, *Encephalitozoon hellem*, *Encephalitozoon intestinalis*, *Encephalitozoon romaleae*, *Nematocida parisii*, and *Nosema ceranae*

^c^Based on genomes of several species of trichuroid nematodes. Note that we did not find Pex1 and Pex6 in the plant pathogens *Globodera pallida*, *Meloidogyne floridensis*, and *Meloidogyne incognita*

^d^Based on the genomes of *Clonorchis sinensis*, *Echinococcus granulosus*, *Echinococcus multilocularis*, *Schistosoma mansoni*, and *Schistosoma haematobium*. A similar set was found for *Hymenolepis microstoma* and *Schistosoma japonicum*, but we did not identify a Yta7 sequence. However, Pex1 and Pex6 sequences were found for the free-living platyhelminthes species *Macrostomum lignano*, *Schmidtea mediterranea*, and *Girardia tigrina*. A partial EST sequence of Pex1 was found for *Dugesia japonica*

### The evolutionary history of the peroxins Pex1 and Pex6 reveals independent losses of peroxisomes in different lineages

It had been noticed earlier that some parasitic protists do not possess peroxisomes, such as the amitochondriate eukaryotes *Encephalitozoon cuniculi* (Microsporidia), *Giardia lamblia* (Diplomonads), *Entamoeba histolytica* (Archamoebae), and *Trichomonas vaginalis* (Trichomonads) as well as several apicomplexans [40, 66, 70, 71]. This assessment is based on the absence of the two peroxins Pex1 and Pex6, among several other factors, which are essential for importing soluble proteins into the lumen of peroxisomes. Our research confirmed this assessment (Table 1), but our broader analysis brought to light some additional aspects: we found that all inspected Microsporidia and Archamoebae genomes do not encode for Pex1 and Pex6, corroborating the loss of peroxisomes in these lineages. We were also unable to find Pex1 and Pex6 in the genome of the anaerobic heterokont *Blastocystis hominis*, suggesting that this intestinal parasite does not have peroxisomes. Furthermore, we did not find Pex1 and Pex6 in many apicomplexans, corroborating earlier accounts [71]. However, we found both genes to be present in coccidians including *Eimeria* and *Toxoplasma*. Both factors are also present in the genome of the chromerids *Chromera velia* and *Vitrella brassicaformis*, which are photosynthetic relatives of apicomplexan parasites [72].

Recently, the absence of key peroxisomal markers in several genomes of parasitic flatworm were reported [73, 74]. Indeed, we did not find Pex1 and Pex6 to be present in the genomes of parasitic Platyhelminthes. However, we found these peroxins in the genome of several free-living flatworms, suggesting that only parasitic species have lost peroxisomes. We did not find Pex1 and Pex6 in the genome of the trichuroid nematodes *Trichinella* and *Trichuris*. This raises the possibility that these nematode lineages might have lost their peroxisomes as well. Furthermore, we did not find both peroxins in the free-living tunicate *Oikopleura dioica*. Similar observations have been reported recently [75]. However, we cannot rule out that the observed lack of peroxins in these organisms is caused by incomplete genome sequencing. In summary, we found recurring losses of the peroxins Pex1 and Pex6 that seem to correlate with the absence or reduction of peroxisomes in these lineages.

### SELMA, a co-opted ERAD machinery

In our analysis, we came across an already established multiplication of Cdc48 that has occurred only in eukaryotic lineages that have engulfed a red algae. The secondary endosymbiosis of another eukaryotic cell, followed by several reductions into a so-called red complex plastid, has taken place in cryptophytes, alveolates, stramenopiles, and haptophytes (CASH) lineages (reviewed in [76–80]). In these lineages, an ERAD system has been co-opted for the transport of proteins across the second-outermost membrane of the symbiotic red algae into the periplastidial compartment (PPC), which can be traced back to the symbiont’s former cytoplasm. Within the PPC, the endosymbiont’s former primary plastid is surrounded by the two innermost membranes, which are homologous to the two membranes of primary plastids. The co-opted import machinery is referred to as symbiont-specific ERAD-like machinery (SELMA). Like the ERAD machinery, SELMA consists of several different proteins acting together with Cdc48, which acts as the central motor; these proteins are referred to as sCdc48 proteins.

**Fig. 5:**
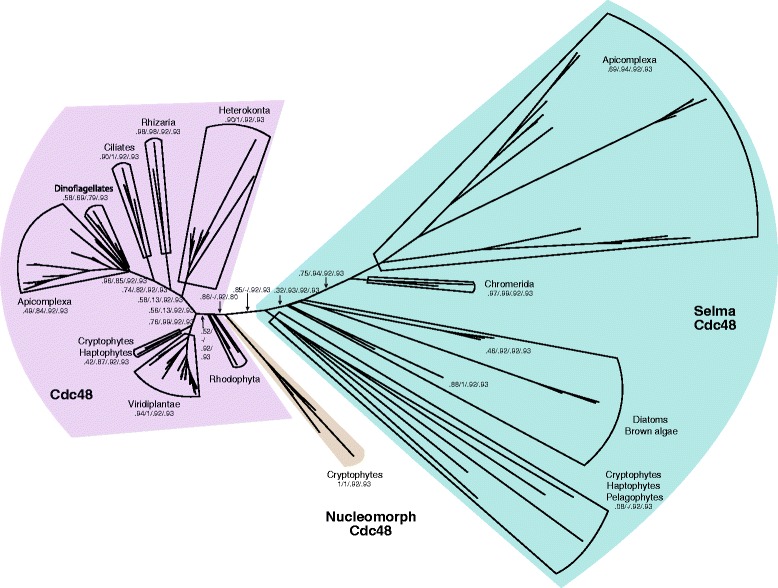
The evolutionary tree of Cdc48 supports the common ancestry of cryptophytes, alveolates, stramenopiles, and haptophytes (CASH lineages). The tree was calculated from the alignment of 687 Cdc48 sequences from a subset of eukaryotic species comprising archaeplastida and the CASH lineages. Statistical support values are annotated at selected inner edges. The tree splits into two main subsections. One subtree contains cytosolic Cdc48 working in the endoplasmic reticulum-associated protein degradation (ERAD) system. The other subtree includes a Cdc48 variant that transports proteins across the second-outermost membrane into the periplastidal compartment, a process referred to as symbiont-specific ERAD-like machinery (SELMA). The branching patterns supports the idea that SELMA Cdc48 is from a common red algal origin in CASH lineages. Note that the Cdc48 encoded by the nucleomorph of cryptophytes is more closely related to the ERAD Cdc48 of red algae. Although the sequences of SELMA Cdc48 diverged rapidly, its subtree still generally reflects the evolutionary relationships of the species. However, in the SELMA Cdc48 subtree, the missing lineages are those that have apparently lost their complex red plastid. Examples are ciliates, dinoflagellates, oomycetes, and cryptosporidians (Additional file 9: Table S3). All four nucleomorphs of cryptophyte algae contain genes for Cdc48 and nuclear VCP-like (NVL) [85], which is known to be involved in ribosome biogenesis. As the entire translation machinery is encoded in the nucleomorph, NVL might play a role in this process. Note that in previous studies, the nucleomorph-encoded Cdc48 and NVL were annotated as Cdc48a and b, respectively [85]. The nucleomorphs do not encode for N-ethylmaleimide sensitive factor (NSF) though, indicating that the red algae endosymbiont does not contain its own endomembrane system [76]

In order to take a closer look at Cdc48’s phylogenetic distribution, we calculated a tree of Cdc48 from CASH lineages and archaeplastida. In agreement with previous studies [41, 81–83], all SELMA Cdc48 sequences form a clear subtree that is well separated from ERAD Cdc48 sequences (Fig. 5). Notably, the SELMA subtree is nested within the ERAD Cdc48s of red algae. A similar branching pattern has been reported for other components of the SELMA machinery [82]. Together, these facts corroborate the idea that the SELMA machinery in CASH lineages evolved only once, probably through a secondary endosymbiosis with a red algae. The ERAD machinery of the engulfed red algae was then co-opted to function as an import system into the cytosol of the red algae [76–80].

### The *C*-terminal HbYX motif of Cdc48

In most lineages, SELMA components are encoded in the host nucleus, which is a good example of endosymbiotic gene transfer. SELMA components carry an *N*-terminal signal sequence and are thought to reach their destination through the host cell’s ER, which is connected with the outermost membrane of the red algae. Interestingly, the host genome of the cryptophyte *Guillardia theta* encodes for two Cdc48 variants, one of which, sCdc48, carries an *N*-terminal signal sequence. According to our phylogenetic analysis, one sCdc48 from *G. theta* is a SELMA component, while the other Cdc48 is working in the ERAD machinery of the host cell. A third Cdc48, a SELMA Cdc48, is encoded by the so-called nucleomorph, which is a residual cell nucleus of the engulfed red algae endosymbiont within the PPC of cryptophytes [84] (Additional file 9: Table S3). Therefore, it seems that some transfer of genetic material to the host genome took place in cryptophytes but it is complete in the other lineages containing red algae.

Nevertheless, this observation raises the question why *G. theta* requires two different variants of Cdc48 in the PPC, both of which can be traced back to an engulfed red algae, one encoded by the nucleomorph and one encoded by the host genome. Interestingly, *G. theta* is not the only species that possesses two sCdc48 variants. In fact, the genome of haptophytes and plastid-bearing heterokonts usually encodes for two sCdc48 variants as well, whereas plastid-bearing apicomplexans and chromerids have only one variant. When we inspected the sequences more closely, it turned out that most species with two sCdc48 variants have one type that still bears a so-called HbYX motif at the *C*-terminus, though the second variant seems to have lost this motif. Unfortunately, the sequence of sCdc48 from *G. theta* does not seem to be complete at the *C*-terminal end. The *C*-terminal HbYX motif is usually highly conserved in eukaryotic Cdc48 (see Additional file 3: Figures S2 and Additional file 6: Figures S4) and is present even in archaeal VAT. This motif is thought to enable Cdc48 to attach directly to the 20S core protease machinery of the proteasome. A similar docking motif is found in the subunits of the 19S regulatory cap of the proteasome. In fact, it has been shown that Cdc48, independent of the 19S regulatory subunit, is able to dock directly onto the 20S particle and to release unfolded substrates into its compartment. This is consistent with the idea that the regulatory proteasome subunits are phylogenetically related to Cdc48 [19, 20, 36, 39].

Could it be that the difference in the C-terminal tail region reflects a division of labor between the two SELMA Cdc48 variants? While one might work on importing proteins into the PPC, the other one might interact with the proteasome to eliminate proteins that are not properly folded. A similar idea has been put forward before [76]. At present, there is no clear answer to this question, as our understanding of the composition of the periplastidial compartment in different lineages and its interchange with the host cell is only beginning to emerge. It seems that cryptophytes like *G. theta* resemble a more ancestral state in the secondary endosymbiosis of a red algae. Its nucleomorph, the remnant nucleus of the engulfed red algae, encodes for a complete repertoire for assembling both subunits of the proteasome. Apparently, the ability to digest proteins that are not properly folded after synthesis has been maintained in the PPC of *G. theta*. The same set of proteins is encoded in the nucleomorph of two other cryptophyte algae, *Hemiselmis andersenii* and *Cryptomonas paramecium*; however, remarkably, the entire set for protein degradation has been lost in the nucleomorph of *Chroomonas mesostigmatica* [85]. At present, it cannot be excluded that these genes have been transferred to the host genome, possibly similar to the situation in the haptophyte *Emiliania huxleyi*, where no proteasome subunits with a signal sequence have been found, although *E. huxleyi* has two sCdc48 variants, one with and one without the HbYX motif.

The situation in plastid-bearing heterokonts is different: their PPC contains only the 20S core protease subunits, which are encoded in the host genome and are transferred to the PPC via a signal peptide [86–88]. Without the 19S cap, the presence of a Cdc48 in the PPC that is able to hand over unfolded substrate to the 20S core proteasome might be important.

No degradation machinery seems to be contained in the PPC or apicoplast of chromerids and plastid-bearing apicomplexans. Their genomes do not encode for proteasome subunits with signal peptides and, at the same time, they encode for only one Cdc48 variant, which is targeted into the periplastidial compartment. In these organisms, material targeted to their vestigial plastid is first synthesized into the lumen of the host ER. As the ER is equipped with chaperones and an ERAD system, transport vesicles might contain only properly folded proteins [89].

## Conclusions

Cdc48, the founding member of a versatile protein family with two AAA ATPase domains in tandem, is one of the most abundant proteins in eukaryotic cells [27]. It is an essential factor that is involved in a large number of different cellular processes. Most of the protein is localized to the cytosol and is often associated with different organelles, whereas another fraction is found in the nucleus. A common theme in its different activities is that Cdc48 extracts ubiquitylated proteins from membranes or complexes and delivers the substrates to the proteasome, although proteasome-independent processes have been described as well. Members of the Cdc48 family form hexameric rings that can undergo coordinated movements driven by ATP hydrolysis.

Our phylogenetic analysis substantiates the idea that the Cdc48 family has expanded and diversified during the rise of eukaryotes from their prokaryotic ancestors. During this pivotal transition, the complex intracellular organization of eukaryotic cells and their various membrane-enclosed organelles with separated metabolic activities evolved. The evolutionary history of the Cdc48 reflects this transition, as several of the novel family members have a narrow spectrum of activities, acting at distinct organelles of the eukaryotic cell, whereas the founding member, Cdc48, has a broad spectrum of activities. It is not known yet whether archaebacterial Cdc48/VAT also has a broad spectrum of activities, thus a direct comparison is not possible, although Cdc48/VAT was found to be essential as well [30]. Our analysis revealed that the LECA was probably equipped with eight distinct Cdc48 family members. This corroborates the notion that the LECA was a fairly sophisticated cell with a nucleus, peroxisomes, and probably all compartments of the endomembrane system.

We detected traces of ancient duplications within the Cdc48 family that probably reflect changes in the subcellular organization that occurred before the LECA. For example, the two peroxins Pex1 and Pex6 arose by gene duplication before the rise of the LECA. They work together in a hetero-hexameric ring with alternating subunits to import proteins into the matrix of peroxisomes. This process resembles the role of Cdc48 during the export of proteins from the ER. Clearly, therefore, the split between the two peroxins must have occurred later than the split between peroxins and Cdc48. The two peroxins and also NSF and Yta7 have diverged greatly, whereas others, particularly Cdc48, have changed only somewhat during the family’s expansion, possibly because Cdc48 remained versatile and interacted with many different cofactors. However, their different evolutionary trajectories render it challenging to deduce in which order or at which point in time the different family members evolved in the evolution of the pre-LECA. Future discoveries may be able to shed more light on this pivotal transition.

Interestingly, some family members have been lost independently in different lineages, for example, Pex1 and Pex6 reflecting the loss of peroxisomes. We also came across an intriguing multiplication of Cdc48 that took place in a eukaryotic lineage that has engulfed a former free-living red algae. The Cdc48 inherited from the red algae has been re-used for the transport of proteins across the second-outermost membrane of the symbiotic red algae. This shows that major changes in cellular compartments can be reflected in the evolutionary history of the Cdc48 family. Although we observed losses of different family members in different lineages, our analysis also revealed that three family members, Cdc48, NVL, and NSF, are maintained throughout all lineages, suggesting that they constitute the minimal set of a eukaryotic cell. Cdc48 and NVL are important for protein homeostasis, whereas NSF fuels vesicle trafficking between the different organelles of the vast eukaryotic endomembrane system. Clearly, without NSF, the eukaryotic cell would lose one of its defining characteristics. Not surprisingly, the only loss of NSF seems to have occurred in the genome of the red algae endosymbiont, the nucleomorph, of *G. theta*, which does not contain an endomembrane system, let alone vesicle trafficking machinery [90]. Under these extreme conditions, the remnant of a eukaryotic cell has been stripped of all but one compartment, the photosynthetic plastid. Nevertheless, how did the vesicle trafficking machinery of the eukaryotic cell evolve without having a dedicated machinery to segregate SNARE complexes already in place? Very probably, at the onset of the emergence of the endomembrane system, a broad-range tandem AAA ATPase like Cdc48 sufficed to disassemble SNARE proteins and NSF evolved afterwards. For example, key elements of the D2-domain of NSF has changed drastically in comparison to Cdc48, suggesting that NSF has lost the ability to hand over the substrate to the proteasome, which is an unnecessary capacity for protein recycling machinery.

## Methods

### Sequence collection and alignment

Initially, we collected a core set of 600 sequences from various eukaryotic and archaeal species of the Cdc48 family that had been established earlier [19]. Initial alignments of the tandem AAA domains were created using MUSCLE [91]. We used secondary structure predictions (e.g. PHD/PHDpsi [92], PSIPRED [93], and Jpred4 [94]), to refine the alignments. The alignments were further improved by incorporating information from the 3D structures of the D2-domain of NSF (1NSF [47], 1D2N [46]), NVL (2X8A), Cdc48 (1R7R [95] and 3CF3 [43]). We used Phyre2 [96] to generate structure predictions for the AAA domains without available 3D structures. From this optimized alignment, we removed columns with more than 50 % gaps and sequences containing more than 50 % gap characters. From the final alignment, we extracted the core motifs of the AAA domains (D1 and D2). By using the profile-profile alignment option in MUSCLE, the two domain alignments were joined into one general AAA domain alignment. To better assess the presence of conserved domains and their arrangement within the collected sequence dataset we used SMART [97], PFAM [98], and CBS [99, 100].

### Classification

To identify subfamilies within the alignment of AAA domains, we used two methods. Firstly, we used CLANS [42], which uses the Basic Local Alignment Search Tool (BLAST) [101] and a subsequent similarity analysis to identify subtypes. Using the implemented network clustering method and different E-value cut-offs, we constructed a hierarchical representation of the collected D-domains. Secondly, we employed phylogenetic reconstruction (see below) to generate an evolutionary classification of the D-domains. In a final step, we reconciled the hierarchies from the two approaches, resulting in a unified classification containing 18 distinct subgroups of the D-domains of the Cdc48 family. We used the HMMER package [102] with standard settings and calibration to train a HMM for each of the subgroups. These HMMs were used to search the National Center for Biotechnology Information (NCBI) RefSeq database (http://www.ncbi.nlm.nih.gov/refseq/). With this extended dataset, we refined our hierarchical classification and further re-trained the HMMs. These models were then used to supplement the dataset by scanning various other sequence resources (Additional file 10: Table S4).

To ensure the high quality of our sequence collection, we visually inspected all predicted AAA domains before incorporating them. During this verification step, we ranked predicted HMM matches by significance (E-value or bit score). The vast majority of identified domains matched one subgroup significantly better than any other (see Additional file 1: Figure S1). However, for a few sequences, ambiguous subgroup affiliations were predicted. To obtain a better understanding of the subgroup affiliation of such sequences, we used Blast and pairwise alignments against our sequence dataset. This approach helped us to refine the affiliation of more divergent sequences and to identify sequences of low quality (see [103] for a detailed discussion of this issue). This refinement process was iteratively continued until no further increase in quality was observed.

### Phylogenetic reconstruction

For phylogenetic reconstructions, we used a combination of three different programs (IQ-TREE [104, 105], Randomized Accelerated Maximum Likelihood (RAxML) [106], and Phylogenetic estimation using Maximum Likelihood (PhyML) [107]). To be able to calculate the best trees, we first used IQ-TREE to estimate best model and model parameters. For all trees, the LG matrix [108] with gamma distribution for rate heterogeneity was found to be the most appropriate model. We executed IQ-TREE with 1000 rapid bootstrap replicates. PhyML was set to start with 20 random start trees and 1000 bootstrap replicates. Additionally, we used Subtree Pruning and Regrafting transformations and a random seed of 9. For RAxML, we again chose a random seed of 9 and 1000 bootstrap replicates. We then used RAxML to estimate site-wise log-likelihoods for all calculated trees and Consel [109] to estimate an Approximately Unbiased (AU) ranking. The highest-ranking tree was taken as a reference. Again making use of Consel, we corrected the support values of the different bootstrap replicates from RAxML and PhyMl using the AU test. IQ-TREE has a built-in correction and no further adjustment was necessary. Finally, as an additional and more independent confidence estimator, we used TREE-PUZZLE [110] to run likelihood-mapping [111] on the best tree. The main edges in all trees are annotated in the following order: likelihood-mapping/IQ-TREE support/RAxML support/PhyML support. The resulting trees are available in Nexus format from our AAA Database web server (see below).

### A web server for access to our results and the de novo classification of the Cdc48 protein family

We have implemented a web-based interface called AAA Database at http://bioinformatics.mpibpc.mpg.de/aaa/index.jsp to provide access to our results. It is divided into three sections. The first section provides access to our collected information, which can be searched for groups, species, and protein names. The second section allows users to submit new sequences to our HMM models. We have implemented the expectation value cut-off to reflect the strict and soft bounds for each family (see Additional file 1: Figure S1). The results display the best four hits and the position of the motif in the alignment. The final section contains the protein alignments and the trees generated for this analysis in Nexus format, which can be analyzed in detail with SplitsTree [112].

## Additional file

Additional file 1: Figure S1. Statistical validation of the AAA domain classification. (A) We used a resampling approach to evaluate the quality of our Hidden Markov Models (HMMs). New models were trained with a random subset of 90 % of the original sequences used to generate each model. We used the other 10 % as the search database with a fixed size of 100,000 sequences. This process was repeated 1000 times and we considered the profile with the best expectation value to be correct. The positive predictive rate (PPR, black, left) and the sensitivity (white, right) are displayed. All models achieved at least 97 % PPR and sensitivity. (B-D) The Cdc48 family is part of a superfamily of classical AAA proteins that also includes proteasome subunits, metalloproteases, meiotic ATPases, and BCS1 [14]. As all our models were trained using Cdc48 AAA domain sequences, non-Cdc48 AAA domain sequences should be a much weaker fit to these models. To evaluate the specificity of our HMMs, we tested the extent to which our models also recognized non-Cdc48 AAA domains. For this, we selected approximately 1800 sequences from the larger family of classical AAA proteins and scanned these sequences with our models. The results are shown as box plots, including the 5 % and 95 % percentiles as whiskers. The plots show the scores (negative logarithm of the expectation value) of our models for the predictions of (B) Cdc48 sequences and (D) non-Cdc48 sequences. We used the different E-value distributions to define the cut-offs for the confidence of our Cdc48 AAA domain predictions. The 5 % percentile of the expectation value distribution in (B) was used as a ‘strict’ cut-off, whereas the 95 % percentile of the expectation value distribution from (D) served as a ‘soft’ cut-off. An overview of the ‘strict’ versus ’soft’ cut-offs for all Cdc48 domain models are displayed in (C). SPAF.d1 is the only model that reveals a lower ‘strict’ than ‘soft’ cut-off. The ‘strict’ cut-off for this model seems especially low, whereas the ‘soft’ cut-off is in a similar range to most other models. Plotting the scores of predictions on Cdc48 sequences for each AAA domain model reveals that most graphs have a logarithmic characteristic, whereas SPAF.d1 follows a linear trend (data not shown). This indicates a higher degree of diversity within this domain. To uphold the quality of our predictions we decided to use the higher ‘soft’ cut-off as the ‘strict’ cut-off for SPAF.d1 as well. (AI 4 mb)
Additional file 2: Table S1. Repertoire of the Cdc48 family members in selected species representing the different major eukaryotic lineages. The eight different Cdc48 family members are present in most eukaryotic lineages, suggesting that these proteins were present in the last eukaryotic common ancestor (LECA). A filled black circle indicates the presence of the family member. A filled blue circle denotes that the factor is encoded by the nucleomorph. (DOCX 44 kb)
Additional file 3: Figure S2. Structural elements of the tandem D-domains of Cdc48. The two D-domains of Cdc48 are formed by two subdomains. (A) The N-terminal αβ subdomain contains various motifs like the Walker A motif (P-loop), the Walker B motif, and the polar Sensor 1 residue, which are important for ATP binding and hydrolysis. The conserved arginine residues at the end of α4, referred to as the Arg finger, are in proximity to the γ-phosphate of the bound ATP in the neighboring subunit. Note that the subunits are active only as hexameric assemblies, a key feature of this protein superfamily. The Cdc48 family belongs to the clade of classical AAA proteins that have a small helical insertion before helix α2 within the Rossman fold [14, 15, 18–20, 22]. The *C*-terminal subdomain is α-helical. A stretch after Helix α7 that was not resolved in the structure is shown as a dashed line. The base of Helix α7 comprises the Sensor 2 region. Both D-domains of Cdc48 possess a conserved GAD motif in this region. The Sensor 2 aspartate of the D1-domain contacts a conserved stretch at the base of the D1-D2 linker and might be important for communication between the two D-domains (Additional file 5: Figure S3). The Sensor 2 aspartate of the D2-domain of Cdc48 interacts with a stretch in front of the *C*-terminal helix and thus might help to position this helix [43, 45]. The tail helix is followed by a *C*-terminal extension with a penultimate HbYX motif (Additional file 6: Figure S4). Usually, this motif is flanked by a stretch of three negatively charged residues. In animals, the tyrosine of the HbYX motif can be phosporylated in vivo [120–122]. The extension serves as binding site for other factors and is also thought to help Cdc48 to dock onto the proteasome. The secondary structure elements are shown according to [18]. (B) Structure of the tandem D-domains of Cdc48 (PDB: 3CF1, [43]). Important structure motifs are colored as in (A). (AI 3 mb)
Additional file 4: Table S2. List of representative species used to calculate the evolutionary trees of the entire Cdc48 family. (DOCX 44 kb)
Additional file 5: Figure S3. A detailed view of the linker region between the D1- and D2-domain. WebLogo representation [119] of (A) the linker region between the D1- and D2-domains of different Cdc48 family members and (B) structure of the linker region between D1- and D2-domain of Cdc48 (PDB: 3CF1 [43]). (AI 7 mb)
Additional file 6: Figure S4. A detailed view of the tail helix region of Cdc48. (A) WebLogo representation [119] of the tail region of different Cdc48 family members. Note that the *C*-terminal HbYX motif of Cdc48 is not maintained in other family members, with the possible exception of Pex1. (B) The structure of the tail region of Cdc48 (PDB: 3CF1 [43]). In the tail helix (in yellow) of Cdc48, the residue Y755 contacts the sensor 1 residue N624. Unfortunately, the structure of the D2 domain of human nuclear VCP-like (NVL, (PDB ID: 2X8A) does not include the tail helix and therefore it cannot be seen whether its tyrosine also interacts with the Sensor 1 asparagine. (AI 5 mb)
Additional file 7: Figure S5. A detailed view of the linker region between the N-domain and D1-domain. (A) WebLogo representation [119] and two conformations of the linker region between the N-domain and D1-domain of Cdc48 in (B) the ADP-bound state and (C) ATP-bound state. This region has been shown to undergo a large conformational change during ATP hydrolysis [54, 55], during which a novel helix is formed. (AI 11 mb)
Additional file 8: Figure S6. Evolutionary tree of the canonical N-domains of different Cdc48 family members. Our analysis revealed that some Cdc48 family members have conserved N-domains. These N-domains are composed of two subdomains Na and Nb. Na is a double-ψβ barrel fold; the Nb domain is an αβ roll. The tree was constructed using the well-conserved homologous N-domains of Cdc48, N-ethylmaleimide-sensitive factor (NSF), Pex1 (N1), Pex6 (N2), Spaf, Spaf-like, and archaeal VCP-like ATPase of Thermoplasma acidophilum (VAT). We did not include the very divergent second N-domains of Pex1 (N2) and Pex2 (N1, see Fig. 1). Note that the N-domains of NSF and of Pex1 (N1) are more divergent than the N domains of the other family members. The different family members are highlighted using the same colors as in Figs. 2 and 4. (AI 293 kb)
Additional file 9: Table S3. Repertoire of Cdc48 in cryptophytes, alveolates, stramenopiles, and haptophytes (CASH lineages) and archaeplastida. A filled black circle denotes the presence of endoplasmic reticulum-associated protein degradation (ERAD) or symbiont-specific ERAD-like machinery (SELMA) Cdc48 in the host cell genome or in the nucleomorph (nm) genome. Note that the host cell genomes of the cryptophytes Chroomonas mesostigmatica, Cryptomonas paramecium, and Hemiselmis andersenii are not currently available (nd). Circles indicate the presence of a photosynthetically active plasmid of red algae origin (red) or of green algae (green); a black circle indicates the presence of a non-photosynthetic plastid remnant. (DOCX 43 kb)
Additional file 10: Table S4. Sequence sources. Data integrated as of 31 December 2015. (DOCX 33 kb)
Additional file 11: Figure S7. Evolutionary trees of the individual AAA domains of the different Cdc48 family members. The trees were constructed as described in Fig. 2, but did not include sequences from archaea (A and B). In (A), the incomplete D2-domain of Yta7 was included, leading to a change in the branching order for the two D-domains of N-ethylmaleimide-sensitive factor (NSF). (ZIP 1 mb)
Additional file 12: Figure S8. WebLogo representation of the two D-domains of the different Cdc48 family members. Sequence logos were generated from alignments of the D-domains of different Cdc48 family members from more than 500 eukaryotes using WebLogo software [119] (see Fig. 3). The overall height of a stack indicates the sequence conservation at a certain position, whereas the height of symbols within the stack indicates the relative frequency of each amino acid at that position. (AI 11 mb)

## Abbreviations

AAA: ATPases associated with various cellular activities
AU: Approximately unbiased
BLAST: Basic Local Alignment Search Tool
CASH: Cryptophytes, alveolates, stramenopiles, and haptophytes
CLANS: CLuster ANalysis of Sequences
ER: Endoplasmic reticulum
ERAD: ER-associated protein degradation
HMMs: Hidden Markov models
LECA: Last eukaryotic common ancestor
NCBI: National Center for Biotechnology Information
NSF: *N*-ethylmaleimide-sensitive factor
NVL: Nuclear VCP-like
PhyML: Phylogenetic estimation using Maximum Likelihood
PPC: Periplastidial compartment
RAxML: Randomized Accelerated Maximum Likelihood
SELMA: ERAD-like machinery
SNAP: Soluble NSF attachment protein
SNARE: Soluble *N*-ethylmaleimide-sensitive factor attachment protein receptor
VAT: VCP-like ATPase of *Thermoplasma acidophilum*
VCP: Valosin-containing protein
