# Supplementary material for: Shedding light on the expansion and diversification of the Cdc48 protein family during the rise of the eukaryotic cell
Source: BMC Evol Biol. 2016 Oct 18;16:215. doi: 10.1186/s12862-016-0790-1 (PMC5070193; doi:10.1186/s12862-016-0790-1)
Supplement: Additional file 2: Table S1. — Repertoire of the Cdc48 family members in selected species representing the different major eukaryotic lineages. The eight different Cdc48 family members are present in most eukaryotic lineages, suggesting that these proteins were present in the last eukaryotic common ancestor (LECA). A filled black circle indicates the presence of the family member. A filled blue circle denotes that the factor is encoded by the nucleomorph. (DOCX 44 kb) [file 12862_2016_790_MOESM2_ESM.docx]

## Table S1. Repertoire of the Cdc48 family members in selected species representing the different major eukaryotic lineages

The eight different Cdc48 family members are present in most eukaryotic lineages, suggesting that these proteins were present in the last eukaryotic common ancestor (LECA). A filled black circle indicates the presence of the family member. A filled blue circle denotes that the factor is encoded by the nucleomorph.

| ***Major Lineage*** | ***Species*** | ***Cdc48*** | ***NSF*** | ***Pex1*** | ***Pex6*** | ***Spaf*** | ***Spaf-like*** | ***NVL*** | ***Yta7*** |
| --- | --- | --- | --- | --- | --- | --- | --- | --- | --- |
| ***Amoebozoa*** | *Dictyostelium discoideum* | •• | • | • | • | • | • | • | • |
|  | *Polysphondylium pallidum* | •• | • | • | • | • | • | • | • |
| ***Fungi*** | *Saccharomyces cerevisiae* | • | • | • | • | • |  | • | • |
|  | *Batrachochytrium dendrobatidis* | • | • | • | • | • | • | • |  |
|  | *Rhizopus oryzae* | • | • | • | • | • | • | • |  |
|  | *Schizosaccharomyces pombe* | • | • | • | • | • |  | • | •• |
| ***Metazoa*** | *Homo sapiens* | • | • | • | • | • | • | • | •• |
|  | *Nematostella vectensis* | • | • | • | • | • | • | • |  |
|  | *Trichoplax adhaerens* | • | • | • | • | • | • | • | • |
|  | *Drosophila melanogaster* | • | •• | • | • | • | • | • |  |
|  | *Caenorhabditis elegans* | •• | • | • | • | • |  | • | • |
| ***Archaeplastida*** | *Ostreococcus tauri* | • | •• | • | • | • | • | • | • |
|  | *Chlamydomonas reinhardtii* | • | • | • | • | • | • | • |  |
|  | *Oryza sativa* | •• | • | • | • | • | •• | • | • |
|  | *Arabidopsis thaliana* | ••• | • | • | • | • | • | • | • |
|  | *Cyanidioschyzon merolae* | • | • | • | • | • | • | • | • |
| ***Excavata*** | *Trichomonas vaginalis* | • | • |  |  | • | • | • |  |
|  | *Leishmania major* | • | •• | • | • | • |  | • | • |
|  | *Trypanosoma brucei* | • | •• | • | • | • |  | • | • |
|  | *Naegleria gruberi* | •• | •• | • | • | • | • | • | • |
| ***Hetero-konta*** | *Phytophthora infestans* | •• | • | • | • | • | • | • | • |
|  | *Ectocarpus siliculosus* | •••• | •• | • | • | • | • | • | • |
| ***Perkin-sozoa*** | *Perkinsus marinus* | •• | • | • | • | • |  | • |  |
| ***Ciliates*** | *Paramecium tetraurelia* | •• | ••• | •• | • | • |  | • |  |
| ***Dinofla-gellata*** | *Symbiodinium minutum* | • | • | • | • | • |  | • |  |
| ***Apicom-plexa*** | *Toxoplasma gondii* | •• | • | • | • | • |  | • |  |
| ***Crypto-phyta*** | *Guillardia theta* | ••• | • | • | • | • | • | •• | • |
| ***Hapto-phyta*** | *Emiliania huxleyi* | ••• | • | • |  | • | • | •• |  |
| ***Rhi-zaria.*** | *Bigelowiella natans* | •• | • | • | • | • |  | •• | • |
