# Supplementary material for: Shedding light on the expansion and diversification of the Cdc48 protein family during the rise of the eukaryotic cell
Source: BMC Evol Biol. 2016 Oct 18;16:215. doi: 10.1186/s12862-016-0790-1 (PMC5070193; doi:10.1186/s12862-016-0790-1)
Supplement: Additional file 4: Table S2. — List of representative species used to calculate the evolutionary trees of the entire Cdc48 family. (DOCX 44 kb) [file 12862_2016_790_MOESM4_ESM.docx]

## Table S2. List of representative species used to calculate the evolutionary trees of the entire Cdc48 family

|  | **Lineage** | **Species name** | **Taxonomic ID (NCBI)** | **Shortname** |
| --- | --- | --- | --- | --- |
| **EUKARYOTA** | Choanoflagellata | *Monosiga brevicollis* MX1 | 431895 | MoBr |
|  | Choanoflagellata | *Salpingoeca sp.* ATCC 50818 | 946362 | SaSp |
|  | Filasterea | *Capsaspora owczarzaki* ATCC 30864 | 595528 | CaOw |
|  | Metazoa | *Amphimedon queenslandica* | 400682 | AmQu |
|  | Metazoa | *Apis mellifera* | 7460 | ApMe |
|  | Metazoa | *Branchiostoma floridae* | 7739 | BrFl |
|  | Metazoa | *Ciona intestinalis* | 7719 | CiIn |
|  | Metazoa | *Daphnia pulex* | 6669 | DaPu |
|  | Metazoa | *Gallus gallus* | 9031 | GaGa |
|  | Metazoa | *Homo sapiens* | 9606 | HoSa |
|  | Metazoa | *Lottia gigantea* | 225164 | LoGi |
|  | Metazoa | *Nematostella vectensis* | 45351 | NeVe |
|  | Metazoa | *Strongylocentrotus purpuratus* | 7668 | StPu |
|  | Fungi | *Batrachochytrium dendrobatidis JEL423* | 403673 | BaDe |
|  | Fungi | *Rhizopus oryzae* RA 99-880 | 246409 | RhOr |
|  | Fungi | *Agaricus bisporus var. bisporus* H97 | 936046 | AgBi |
|  | Fungi | *Puccinia graminis f. sp. tritici* CRL 75-36-700-3 | 418459 | PuGr |
|  | Fungi | *Aspergillus clavatus* NRRL 1 | 344612 | AsCl |
|  | Fungi | *Candida caseinolytica* NRRL Y-17796 | 767744 | CaCas |
|  | Fungi | *Mycosphaerella fijiensis* | 83344 | MyFi |
|  | Fungi | *Phaeosphaeria nodorum* SN15 | 321614 | PhNo |
|  | Fungi | *Schizosaccharomyces pombe* | 4896 | ScPo |
|  | Fungi | *Yarrowia lipolytica* CLIB99 | 284591 | YaLi |
|  | Amoebozoa | *Acanthamoeba castellanii* str. Neff | 1257118 | AcCa |
|  | Mycetozoa | *Dictyostelium discoideum* AX4 | 352472 | DiDi |
|  | Kinetoplastida | *Leishmania infantum* JPCM5 | 435258 | LeIn |
|  | Kinetoplastida | *Trypanosoma cruzi strain* CL Brener | 353153 | TrCr_1 |
|  | Chlorophyta | *Chlamydomonas reinhardtii* | 3055 | ChRe |
|  | Chlorophyta | *Chlorella sp.* NC64A | 310507 | ChSp |
|  | Chlorophyta | *Coccomyxa sp.* C-169 | 574566 | CoSp |
|  | Chlorophyta | *Ostreococcus lucimarinus* CCE9901 | 436017 | OsLu |
|  | Streptophyta | *Arabidopsis thaliana* | 3702 | ArTh |
|  | Streptophyta | *Oryza sativa* Japonica Group | 39947 | OrSa |
|  | Streptophyta | *Physcomitrella patens subsp. patens* | 145481 | PhPa |
|  | Streptophyta | *Selaginella moellendorffii* | 88036 | SeMo |
|  | Cryptophyta | *Guillardia theta* | 55529 | GuTh |
|  | Haptophyceae | *Emiliania huxleyi* CCMP1516 | 280463 | EmHu |
|  | Heterokontophyta | Albugo laibachii Nc14 | 890382 | AlLa |
|  | Heterokontophyta | *Aureococcus anophagefferens* CCMP1984 | 44056 | AuAn |
|  | Heterokontophyta | *Ectocarpus siliculosus* | 2880 | EcSil |
|  | Heterokontophyta | *Phytophthora infestans* T30-4 | 403677 | PhIn |
|  | Heterokontophyta | *Phaeodactylum tricornutum* CCAP 1055/1 | 556484 | PhTr |
|  | Heterokontophyta | *Thalassiosira pseudonana* CCMP1335 | 296543 | ThPs |
|  | Apicomplexa | *Babesia bovis* T2Bo | 484906 | BaBo |
|  | Apicomplexa | *Cryptosporidium parvum* Iowa II | 353152 | CrPa |
|  | Apicomplexa | *Plasmodium falciparum* 3D7 | 36329 | PlFa |
|  | Apicomplexa | *Theileria annulata* strain Ankara | 353154 | ThAn |
|  | Perkinsea | *Perkinsus marinus* ATCC 50983 | 423536 | PerMa |
| **ARCHAEA** | Euryarchaeota | *Aciduliprofundum boonei* T469 | 439481 | AcBo |
|  | Euryarchaeota | *Archaeoglobus fulgidus* DSM 4304 | 224325 | ArFu |
|  | Euryarchaeota | *Ferroglobus placidus* DSM 10642 | 589924 | FePl |
|  | Euryarchaeota | *Ferroplasma acidarmanus* fer1 | 333146 | FeAc |
|  | Euryarchaeota | *Halalkalicoccus jeotgali* B3 | 795797 | HaJe |
|  | Euryarchaeota | *Halobacterium sp.* NRC-1 | 64091 | HalSp |
|  | Euryarchaeota | *Methanocaldococcus vulcanius* M7 | 579137 | MeVul |
|  | Euryarchaeota | *Methanocella paludicola* SANAE | 304371 | MePal |
|  | Euryarchaeota | *Methanococcus aeolicus* Nankai-3 | 419665 | MeAe |
|  | Euryarchaeota | *Methanoculleus marisnigri* JR1 | 368407 | MeMar |
|  | Euryarchaeota | *Methanopyrus kandleri* AV19 | 190192 | MeKa |
|  | Euryarchaeota | *Methanosphaera stadtmanae* DSM 3091 | 339860 | MeSt |
|  | Euryarchaeota | *Picrophilus torridus* DSM 9790 | 263820 | PiTo |
|  | Euryarchaeota | *Pyrococcus furiosus* DSM 3638 | 186497 | PyFu |
|  | Euryarchaeota | *Pyrococcus horikoshii* OT3 | 70601 | PyHo |
|  | Crenarchaeota | *Acidilobus saccharovorans* 345-15 | 666510 | AcSa |
|  | Crenarchaeota | *Caldivirga maquilingensis* IC-167 | 397948 | CaMa |
|  | Crenarchaeota | *Hyperthermus butylicus* DSM 5456 | 415426 | HyBu |
|  | Crenarchaeota | *Ignisphaera aggregans* DSM 17230 | 583356 | IgAg |
|  | Crenarchaeota | *Metallosphaera sedula* DSM 5348 | 399549 | MeSe |
|  | Crenarchaeota | *Sulfolobus islandicus* Y.N.15.51 | 419942 | SuIs |
|  | Crenarchaeota | *Thermofilum pendens* Hrk 5 | 368408 | ThPen |
|  | Thaumarchaeota | *Cenarchaeum symbiosum* A | 414004 | CeSy |
|  | Thaumarchaeota | *Nitrosopumilus maritimus* SCM1 | 436308 | NiMa |
|  | Korarchaeota | *Candidatus Korarchaeum cryptofilum* OPF8 | 374847 | CaKo |
|  | Nanoarchaeota | *Nanoarchaeum equitans* Kin4-M | 228908 | NaEq |
