# Supplementary material for: Shedding light on the expansion and diversification of the Cdc48 protein family during the rise of the eukaryotic cell
Source: BMC Evol Biol. 2016 Oct 18;16:215. doi: 10.1186/s12862-016-0790-1 (PMC5070193; doi:10.1186/s12862-016-0790-1)
Supplement: Additional file 9: Table S3. — Repertoire of Cdc48 in cryptophytes, alveolates, stramenopiles, and haptophytes (CASH lineages) and archaeplastida. A filled black circle denotes the presence of endoplasmic reticulum-associated protein degradation (ERAD) or symbiont-specific ERAD-like machinery (SELMA) Cdc48 in the host cell genome or in the nucleomorph (nm) genome. Note that the host cell genomes of the cryptophytes Chroomonas mesostigmatica, Cryptomonas paramecium, and Hemiselmis andersenii are not currently available (nd). Circles indicate the presence of a photosynthetically active plasmid of red algae origin (red) or of green algae (green); a black circle indicates the presence of a non-photosynthetic plastid remnant. (DOCX 43 kb) [file 12862_2016_790_MOESM9_ESM.docx]

## Table S3. Repertoire of Cdc48 in cryptophytes, alveolates, stramenopiles, and haptophytes (CASH lineages) and archaeplastida

A filled black circle denotes the presence of endoplasmic reticulum-associated protein degradation (ERAD) or symbiont-specific ERAD-like machinery (SELMA) Cdc48 in the host cell genome or in the nucleomorph (nm) genome. Note that the host cell genomes of the cryptophytes *Chroomonas mesostigmatica*, *Cryptomonas paramecium*, and *Hemiselmis andersenii* are not currently available (nd). Circles indicate the presence of a photosynthetically active plasmid of red algae origin (red) or of green algae (green); a black circle indicates the presence of a non-photosynthetic plastid remnant.

| ***Phylum*** | **Lineage** | **Species** | **Plastid** | **ERADCdc48** | **SELMA CDC48** | **nm**  **Cdc48** |
| --- | --- | --- | --- | --- | --- | --- |
| ***ALVEOLATA*** | Apicomplexa | *Plasmodium berghei* | 🞆 | • | • |  |
|  |  | *Plasmodium chabaudi* | 🞆 | • | • |  |
|  |  | *Plasmodium falciparum* | 🞆 | • | • |  |
|  |  | *Plasmodium knowlesi* | 🞆 | • | • |  |
|  |  | *Plasmodium vivax* | 🞆 | • | • |  |
|  |  | *Plasmodium yoelii* | 🞆 | • | • |  |
|  |  | *Babesia bovis* | 🞆 | • | • |  |
|  |  | *Babesia equi* | 🞆 | • | • |  |
|  |  | *Babesia microti* | 🞆 | • | • |  |
|  |  | *Babesia bigemina* | 🞆 | • | • |  |
|  |  | *Theileria orientalis* | 🞆 | • | • |  |
|  |  | *Theileria parva* | 🞆 | • | • |  |
|  |  | *Theileria annulata* | 🞆 | • | • |  |
|  |  | *Eimeria brunetti* | 🞆 | • | • |  |
|  |  | *Eimeria maxima* | 🞆 | • | • |  |
|  |  | *Eimeria mitis* | 🞆 | • | • |  |
|  |  | *Eimeria necatrix* | 🞆 | • | • |  |
|  |  | *Eimeria tenella* | 🞆 | • | • |  |
|  |  | *Eimeria falciformis* | 🞆 | • | • |  |
|  |  | *Hammondia hammondi* | 🞆 | • | • |  |
|  |  | *Neospora caninum* | 🞆 | • | • |  |
|  |  | *Toxoplasma gondii* | 🞆 | • | • |  |
|  |  | *Cryptosporidium hominis* |  | • |  |  |
|  |  | *Cryptosporidium muris* |  | • |  |  |
|  |  | *Cryptosporidium parvum* |  | • |  |  |
|  |  | *Gregarina niphandrodes* |  | • |  |  |
|  | Chro-merids | *Chromera velia* | 🞆 | • | • |  |
|  |  | *Vitrella brassicaformis* | 🞆 | • | • |  |
|  |  | *Perkinsus marinus* |  | •• |  |  |
|  |  | *Symbiodinium minutum* |  | ••• |  |  |
|  | Ciliates | *Paramecium tetraurelia* |  | •• |  |  |
|  |  | *Tetrahymena thermophila* |  | • |  |  |
|  |  | *Oxytricha trifallax* |  | • |  |  |
| ***HETEROKONTA*** |  | *Blastocystis hominis* |  | • |  |  |
|  |  | *Phytophthora capsici* |  | •• |  |  |
|  |  | *Phytophthora infestans* |  | •• |  |  |
|  |  | *Phytophthora ramorum* |  | •• |  |  |
|  |  | *Phytophthora sojae* |  | •• |  |  |
|  |  | *Albugo laibachii* |  | •• |  |  |
|  |  | *Saprolegnia parasitica* |  | •• |  |  |
|  |  | *Fragilariopsis cylindrus* | 🞆 | • | •• |  |
|  |  | *Phaeodactylum tricornutum* | 🞆 | • | •• |  |
|  |  | *Thalassiosira pseudonana* | 🞆 | •• | •• |  |
|  |  | *Ectocarpus siliculosus* | 🞆 | •• | •• |  |
|  |  | *Aureococcus anophagefferens* | 🞆 | • | •• |  |
| ***CRYPTOPHYTA*** |  | *Guillardia theta* | 🞆 | • | • | • |
|  |  | *Chroomonas mesostigmatica* | 🞆 | nd | nd | • |
|  |  | *Cryptomonas paramecium* | 🞆 | nd | nd | • |
|  |  | *Hemiselmis andersenii* | 🞆 | nd | nd | • |
| ***HAPTO-PHYTA*** |  | *Emiliania huxleyi* | 🞆 | • | •• |  |
| ***ARCHAEPLASTIDA*** | Rhodophyta | *Chondrus crispus* | 🞆 | • |  |  |
|  |  | *Cyanidioschyzon merolae* | 🞆 | • |  |  |
|  |  | *Galdieria sulphuraria* | 🞆 | • |  |  |
|  | Green Algae | *Chlorella variabilis* | 🞆 | •• |  |  |
|  |  | *Chlamydomonas reinhardtii* | 🞆 | • |  |  |
