# Supplementary material for: Shedding light on the expansion and diversification of the Cdc48 protein family during the rise of the eukaryotic cell
Source: BMC Evol Biol. 2016 Oct 18;16:215. doi: 10.1186/s12862-016-0790-1 (PMC5070193; doi:10.1186/s12862-016-0790-1)
Supplement: Additional file 10: Table S4. — Sequence sources. Data integrated as of 31 December 2015. (DOCX 33 kb) [file 12862_2016_790_MOESM10_ESM.docx]

## Table S4 - Sequence sources

Data integrated as of 31 December 2015.

| ***Source*** | ***URL*** |
| --- | --- |
| NCBI RefSeq | <http://www.ncbi.nlm.nih.gov/refseq/> |
| Welcome Trust Sanger Institute | <http://www.sanger.ac.uk> |
| Broad Institute | <http://www.broadinstitute.org/> |
| Department of Energy Joint Genome Institute | <http://genome.jgi.doe.gov/> |
| J. Craig Venter Institute | <http://www.jcvi.org/cms/home/> |
| Genoscope: Centre National de Sequencage | <http://www.genoscope.cns.fr/spip/> |
| Social Amoebas Comparative Genome Browser | <http://sacgb.fli-leibniz.de/cgi/index.pl> |
| Dictybase | <http://dictybase.org> |
| Eukaryotic Pathogens Database Resources | <http://eupathdb.org/eupathdb/> |
| Cyanophora Genome Project | <http://cyanophora.rutgers.edu/cyanophora/home.php> |
| Silkworm Genome Database | <http://silkworm.genomics.org.cn/> |
| The Pleurobrachia Genome | <http://neurobase.rc.ufl.edu/pleurobrachia> |
| Mnemiopsis Genome Project | <http://research.nhgri.nih.gov/mnemiopsis/> |
| *Cyanidioschyzon merolae* Genome Project | <http://merolae.biol.s.u-tokyo.ac.jp/> |
